# Wear and Tear, or Hints for the Overworked

**Published:** 1887-05

**Authors:** 


WEAR AND TEAR, OR HINTS FOR THE OVERWORKED. *By* S. WEIR MITCHELL, M.D., LL.D., *Member of the National Academy of Sciences. Fifth edition, pp. 76. 1887. Philadelphia:* J. B. LIPPINCOTT & Co.

Of all that has been written on "American Nervousness," and the various phases of brain exhaustion as they come under the observation of the neurologist, none have appealed so strongly to the common sense of both the lay and professional public, as this little work on "Wear and Tear."

To the physician much of the work appears trite and commonplace, it being written originally for the pages of a popular magazine, and addressed to non-professional readers. This is an added advantage, as it can often be prescribed for patients of this class with more benefit than the more popular bromide and ergot.

Any extended discussion of the peculiar views in this little work is scarcely called for at this time. At the end of fifteen years a fifth edition is called for, and the work is still found to be useful.

